# Supplementary material for: Global risk mapping of highly pathogenic avian influenza H5N1 and H5Nx in the light of epidemic episodes occurring from 2020 onwards
Source: eLife. 2026 Jan 28;14:RP104748. doi: 10.7554/eLife.104748 (PMC12851579; doi:10.7554/eLife.104748)
Supplement: Supplementary file 1. [file elife-104748-supp1.docx]

**Supplementary Table 1: assessment of the predictive performance of ecological niche models to predict present and past ecological suitability maps.** We here report, for each replicate analysis, a measure of the predictive performance of the trained ecological niche through the computation of the area under the receiver operating characteristic (ROC) curve, also simply referred to as “area under the curve” (AUC). (*) refers to AUC estimates obtained when assessing the capacity of models trained on occurrence data < 2020 to predict > 2020 distribution of occurrence data.

|  | **Wild birds** | | | **Domestic birds** | | | | | |
| --- | --- | --- | --- | --- | --- | --- | --- | --- | --- |
|  | **H5Nx** | | | **H5N1** | | | **H5Nx** | | |
|  | < 2020 | > 2020 | >2020* | < 2020 | > 2020 | > 2020* | < 2020 | > 2020 | > 2020* |
| Replicate 1 | 0.899 | 0.793 | 0.700 | 0.832 | 0.812 | 0.769 | 0.833 | 0.791 | 0.755 |
| Replicate 2 | 0.896 | 0.769 | 0.703 | 0.832 | 0.822 | 0.769 | 0.829 | 0.781 | 0.752 |
| Replicate 3 | 0.895 | 0.796 | 0.707 | 0.834 | 0.820 | 0.770 | 0.818 | 0.784 | 0.743 |
| Replicate 4 | 0.894 | 0.773 | 0.705 | 0.843 | 0.820 | 0.769 | 0.828 | 0.804 | 0.751 |
| Replicate 5 | 0.893 | 0.797 | 0.706 | 0.837 | 0.816 | 0.770 | 0.828 | 0.780 | 0.752 |
| Replicate 6 | 0.898 | 0.797 | 0.701 | 0.845 | 0.816 | 0.770 | 0.829 | 0.780 | 0.752 |
| Replicate 7 | 0.897 | 0.798 | 0.700 | 0.835 | 0.816 | 0.769 | 0.827 | 0.775 | 0.750 |
| Replicate 8 | 0.894 | 0.805 | 0.705 | 0.836 | 0.825 | 0.770 | 0.828 | 0.777 | 0.752 |
| Replicate 9 | 0.894 | 0.770 | 0.702 | 0.834 | 0.813 | 0.769 | 0.828 | 0.779 | 0.750 |
| Replicate 10 | 0.894 | 0.775 | 0.703 | 0.835 | 0.823 | 0.769 | 0.832 | 0.795 | 0.754 |

**Supplementary Table 2: relative influence (RI, in %) of each environmental variable in the respective ecological models trained on a specific dataset of occurrence data.** A RI estimate was obtained for each replicate BRT analysis and we here report median as well as first and third quartile RI values.

|  | **Wild birds** | | | |
| --- | --- | --- | --- | --- |
|  | **H5N1** | | **H5Nx** | |
|  | **< 2020** | **> 2020** | **< 2020** | **> 2020** |
| Evergreen deciduous needleleaf trees | - | 1.6 % [1.5-1.6] | 1.6 % [1.3-1.7] | 0.4 % [0.4-0.5] |
| Evergreen broadleaf trees | - | 1.3 % [1.2-1.4] | 0.1 % [0.1-0.1] | 1.0 % [0.8-1.1] |
| Deciduous broadleaf trees | - | 4.1 % [4.0-4.2] | 1.6 % [1.5-1.8] | 6.1 % [5.9-6.2] |
| Mixed and other trees | - | 11.5 % [11.4-11.7] | 5.3 % [4.9-5.4] | 11.4 % [11.3-11.8] |
| Shrublands | - | 2.3 % [2.0-2.3] | 3.2 % [3.0-3.3] | 2.4 % [2.1-2.7] |
| Herbaceous vegetation | - | 8.8 % [8.8-9.0] | 2.8 % [2.5-3.0] | 9.5 % [9.5-9.6] |
| Cultivated and managed vegetation | - | 4.4 % [4.3-4.5] | 4.8 % [4.6-4.9] | 5.8 % [5.4-6.1] |
| Regularly flooded vegetation | - | 0.3 % [0.3-0.3] | 0.1 % [0.1-0.1] | 0.2 % [0.1-0.3] |
| Urban and built-up areas | - | 39.3 % [39.0-39.9] | 54.5 % [53.5-55.0] | 37.5 % [36.5-38.2] |
| Open water areas | - | 22.0 % [21.9-22.3] | 25.5 % [25.3-25.8] | 23 % [22.8-23.3] |
| Distance to water | - | 4.1 % [3.9-4.3] | 0.9 % [0.7-1.0] | 2.8 % [2.4-3.1] |
|  | **Domestic birds** | | | |
|  | **H5N1** | | **H5Nx** | |
|  | **< 2020** | **> 2020** | **< 2020** | **> 2020** |
| Duck pop. density (2010, log) | 18.2 % [17.8-18.4] | 15.7 % [13.3-16.4] | 28.7 % [28.4-28.8] | 12.9 % [12.9-13.5] |
| Extensive chicken pop. density (2015, log) | 4.4 % [4.2-4.5] | 2.3 % [0.5-2.6] | 3.9 % [3.7-4.0] | 2.7 % [2.5-3.4] |
| Intensive chicken pop. density (2015, log) | 8.5 % [8.4-8.7] | 30.4 % [29.6-36.2] | 29.6 % [29.3-30.1] | 43.1 % [40.5-43.4] |
| Human pop. density (2020, log) | 54.9 % [53.9-55.1] | 20.7 % [20.4-21.6] | 15.5 % [15.3-15.7] | 14.9 % [14.8-15.2] |
| Cultivated and managed vegetation | 9.5 % [9.3-9.7] | 16.6 % [16.3-18.5] | 7.1 % [7.0-7.2] | 13.2 % [12.9-13.3] |
| Open water areas | 0.5 % [0.5-0.6] | 1.4 % [0.3-1.5] | 0.0 % [0.0-0.0] | 0.6 % [0.4-0.8] |
| Day LST annual mean | 4.1 % [4.0-4.2] | 12.9 % [9.6-13.3] | 15.4 % [15.1-15.6] | 13.0 % [12.4-13.9] |
