# Supplementary material for: Global risk mapping of highly pathogenic avian influenza H5N1 and H5Nx in the light of epidemic episodes occurring from 2020 onwards
Source: eLife. 2026 Jan 28;14:RP104748. doi: 10.7554/eLife.104748 (PMC12851579; doi:10.7554/eLife.104748)
Supplement: Supplementary file 2. — The three panels successively show the total occurrence records for all H5Nx subtypes, occurrence records for non-H5N1 H5Nx subtypes, and all H5N1 occurrence records. [file elife-104748-supp2.docx]

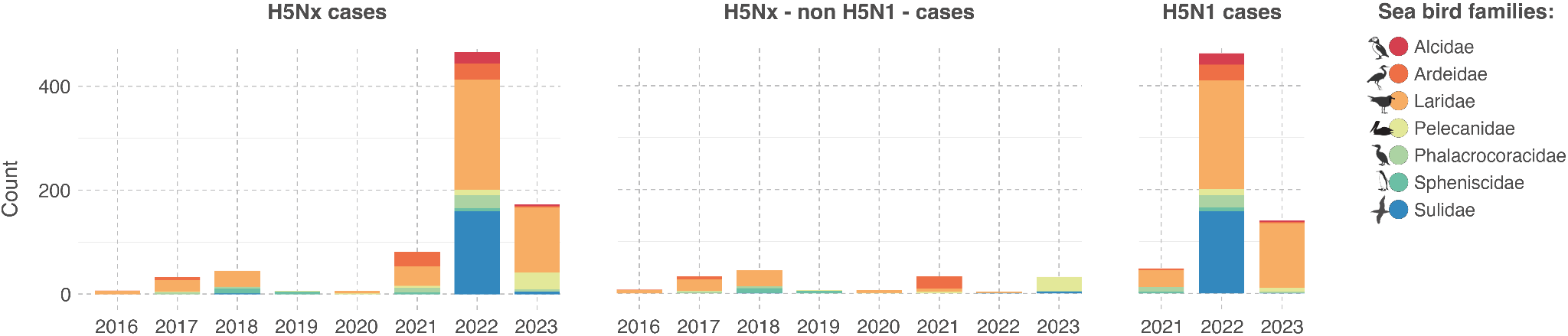


**Distribution of H5Nx and H5N1 occurrence records in sea birds from 2015 to 2023, categorised by bird family**. The three panels successively show the total occurrence records for all H5Nx subtypes, occurrence records for non-H5N1 H5Nx subtypes, and all H5N1 occurrence records.
